# Supplementary material for: Functional imaging of visual cortical layers and subplate in awake mice with optimized three-photon microscopy
Source: Nat Commun. 2019 Jan 11;10:177. doi: 10.1038/s41467-018-08179-6 (PMC6329792; doi:10.1038/s41467-018-08179-6)
Supplement: Supplementary file 1 — Supplementary Information [file 41467_2018_8179_MOESM1_ESM.pdf]

# SUPPLEMENTARY INFORMATION

Functional imaging of visual cortical layers and subplate in awake mice with optimized three-photon microscopy

Yildirim et al.

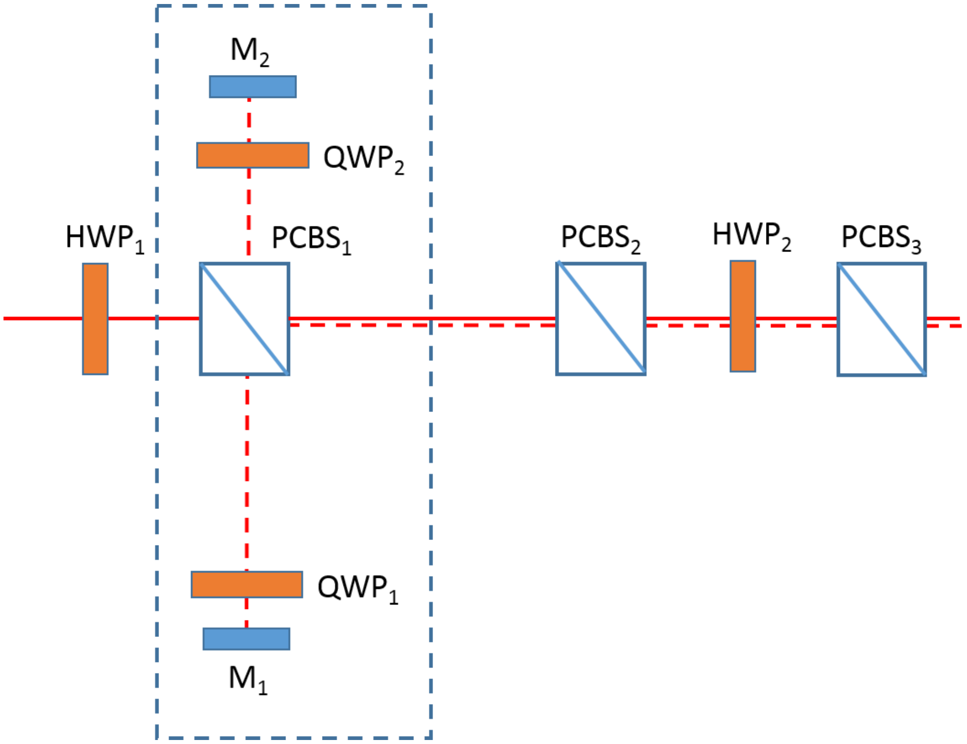


**Supplementary Figure 1. Delay line system for doubling the repetition rate**. . HWP: half-wave plate; QWP: quarter-wave plate; PCBS: polarizing cube beam splitter; M: mirror. The laser power is first equally separated through HWP1 and PCBS1. Then, the transmitted part is transmitted through other optics such as PCBS2, HWP2, and PCBS3. The reflected part's polarization is changed 90 degrees via first with QWP1 and M1, and then QWP2 and M2. The total optical path length of the reflected part is 0.9 m which corresponds to 3 ns separation for these two beams. Then, both beams are recombined.

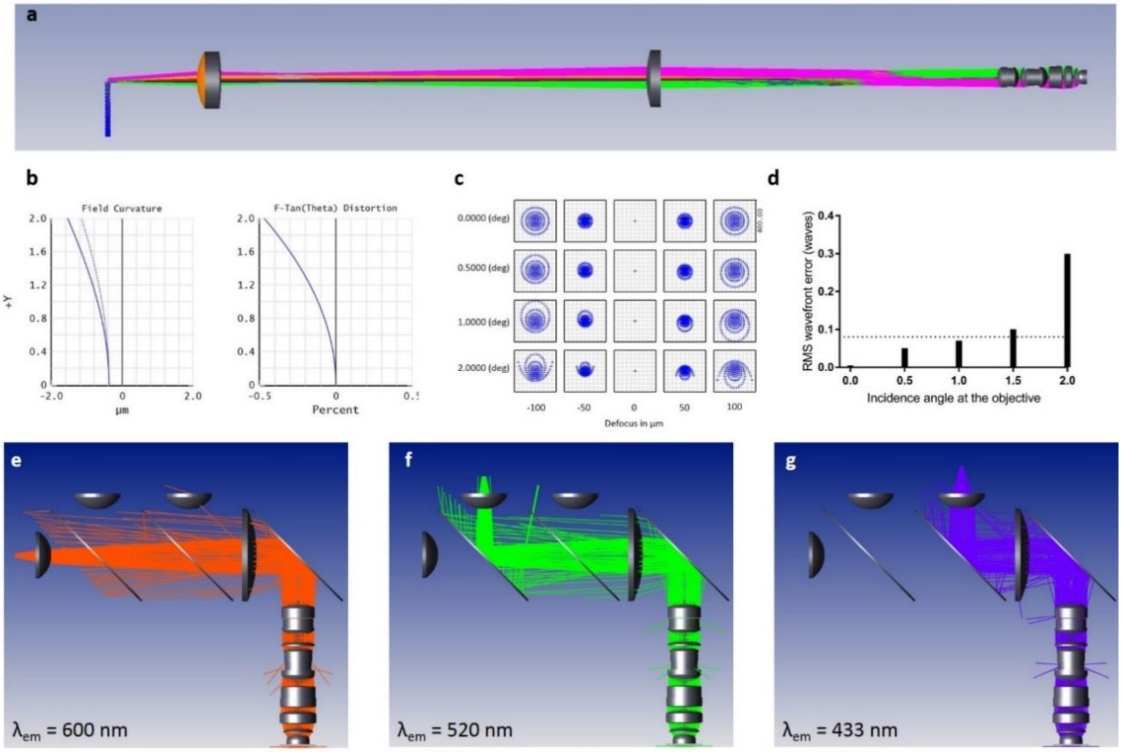


**Supplementary Figure 2. Layout of the excitation and emission part of the microscope**. (a) Zemax model of the excitation beam with one galvo scanner, combination of scan and tube lens, and the objective. Different colors represent 4 different scanning angles ranging from 0° to 10° which correspond to scanning angles of 0° and 2° at the objective back aperture. (b) Field curvature and theta distortion over the entire scanning range on the sample. (c) Spot size matrix through the focal plane for all scanning angle range. (d) RMS wavefront error with varying the incidence angle at the objective. (e) Zemax model of the emission part of the microscope with seawater as a sample having bulk Henyey-Greenstein scattering. The emitted photons ($10^6$) from the focal plane (1 mm deep) are collected by the objective and reflected to the first collection optics lens. Then, emitted photons are collected by their corresponding collection optics depending on the corresponding PMT. Emitted photon collection shown for retrobeads imaging at 600 nm (e), for GCaMP imaging at 520 nm (f), and for THG imaging at 433 nm (g). Retrobeads are retrograde tracers used for anatomical labeling of cell bodies from their target projection zones.

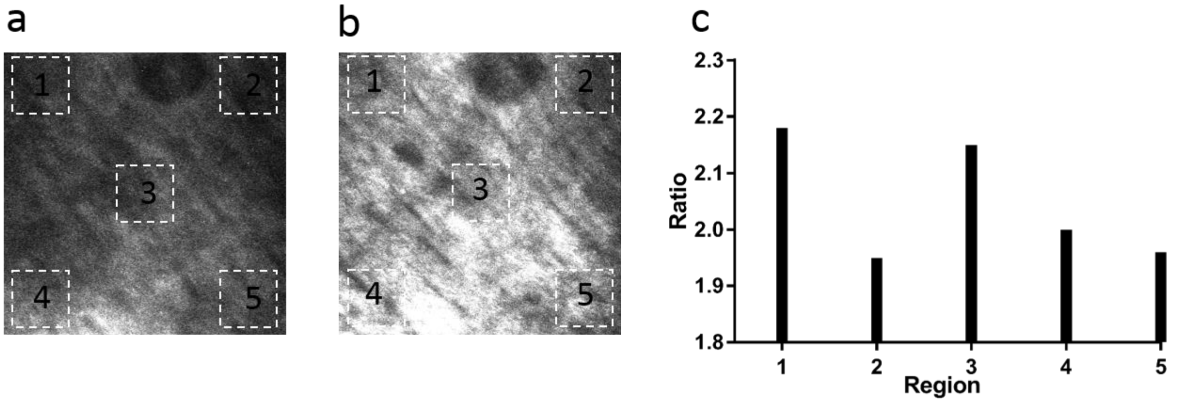


**Supplementary Figure 3. Comparison of two-photon and three-photon microscope objectives from Olympus (25x, 1.05 NA) via imaging axonal tracts in the white matter**. (a) THG imaging of axonal tracts in the white matter with conventional two-photon (2p) objective at 8 mW average laser power, (b) THG imaging of the same field of view in (a) with three-photon (3p) objective at 8 mW average laser power. (c) Ratio of the average signals obtained with 3p and 2p objectives in five different regions of the same field view. Overall improvement with 3p objective is two fold.

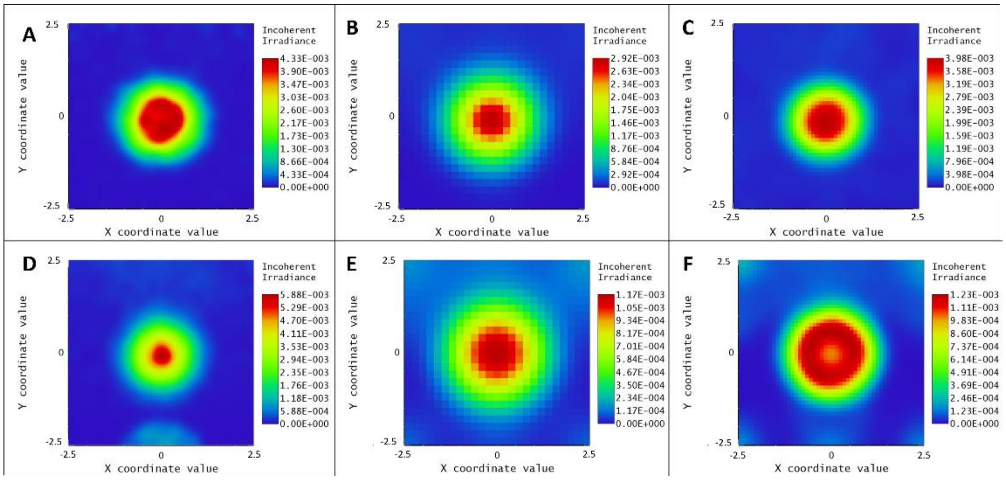


**Supplementary Figure 4. Comparison of one-inch and two-inch optics in the emission path.** Total number of photons become 57815, 53960, and 60291 for THG (A), GCaMP6s (B), and retrobeads (C) PMTs in one-inch design, respectively. On the other hand, 2-inch design corresponds to 117421, 121697, and 119263 photons for THG (D), GCaMP6s (E), and retrobeads (F) PMTs with $10^6$ photons generated at the focal plane.

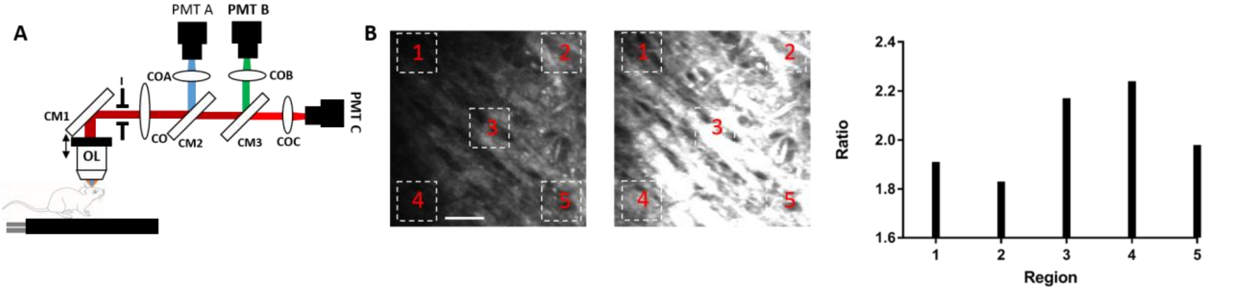


**Supplementary Figure 5. Comparison of one-inch and two-inch size collection optics by placing an iris (I) in front of the collection side of the microscope**. (A) Schematic of the experimental setup modification. See legend of Fig. 1 for abbreviations of optical components. (B) Imaging of axonal tracts in the white matter via THG microscopy with one-inch size iris (left), and with two-inch size iris (right). (C) Ratio of THG signal acquired with two-inch size iris and with one-inch iris. Scale bar is 50 µm.

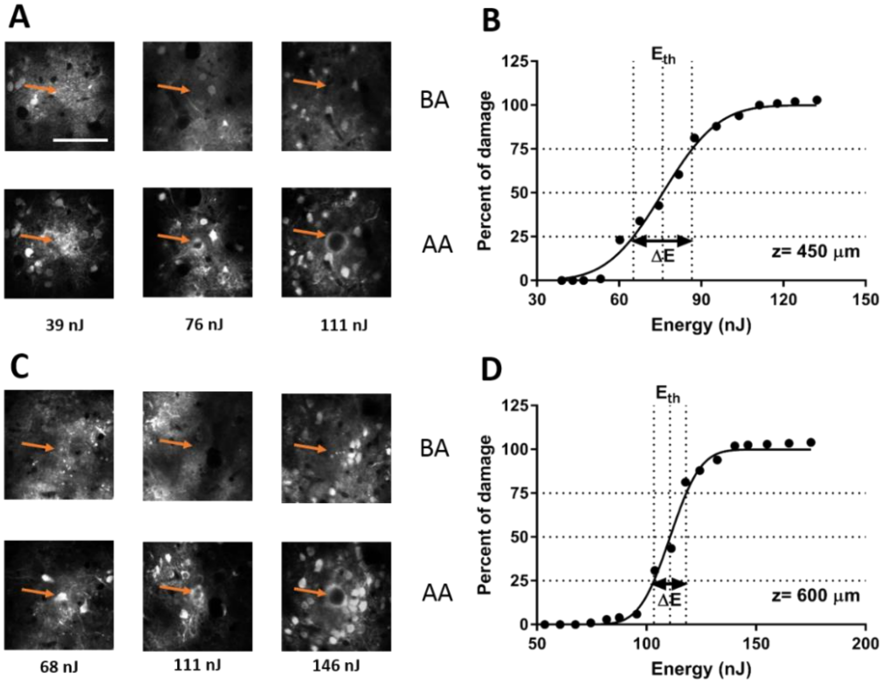


**Supplementary Figure 6. Characterization of optical properties of live mouse cortex via laser ablation.** (A) Representative images before (BA) and after (AA) ablation for three pulse energies (39, 76, and 111 nJ) at 450 µm depth. (B) Determining extinction length via tissue ablation. Percent of damage ranges from 0 to 100% with respect to laser energy on the tissue surface. Threshold energy (Eth) is the energy which results in 50% damage. For ablation at 450 µm depth, Eth is 75.9 nJ. (C) Representative images before (BA) and after (AA) ablation for three pulse energies (68,111, and 146 nJ) at 600 µm depth. (D) Determining extinction length via tissue ablation. Percent of damage ranges from 0 to 100% with respect

to laser energy on the tissue surface. Threshold energy (Eth) is the energy which results in 50% damage. For ablation at 600 µm depth, Eth is 110.7 nJ.

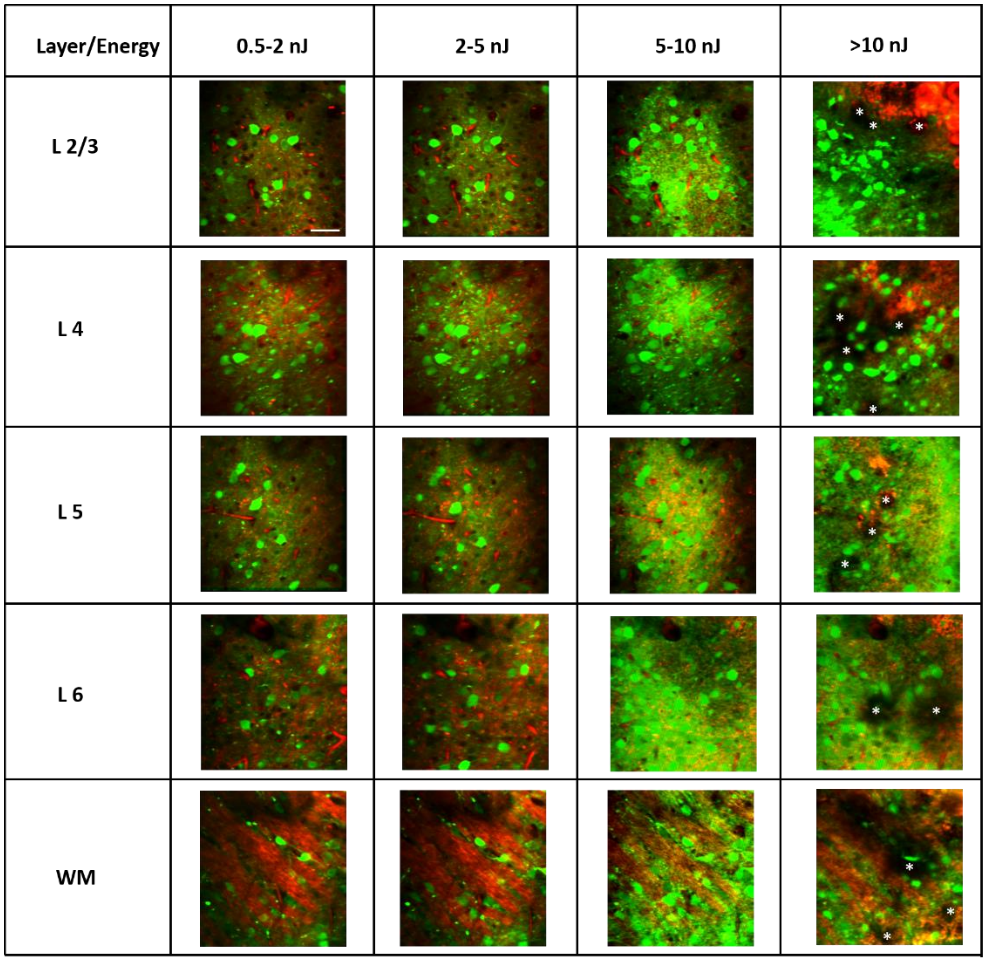


**Supplementary Figure 7. Characterization of evoked neuronal responses at each layer with varying pulse energies on the focal plane**. Representative images at each layer while pulse energies at the focal plane are varied from 0.5 nJ to 15 nJ. No saturation is observed up to 5 nJ pulse (first and second column), whereas GCaMP6s signal is saturated >5 nJ pulse energies (third column). Physical damage is observed for >10 nJ pulse energies (fourth column). Asterisks in the fourth column represent physical damage due to optical breakdown. Scale bar is 50 µm.

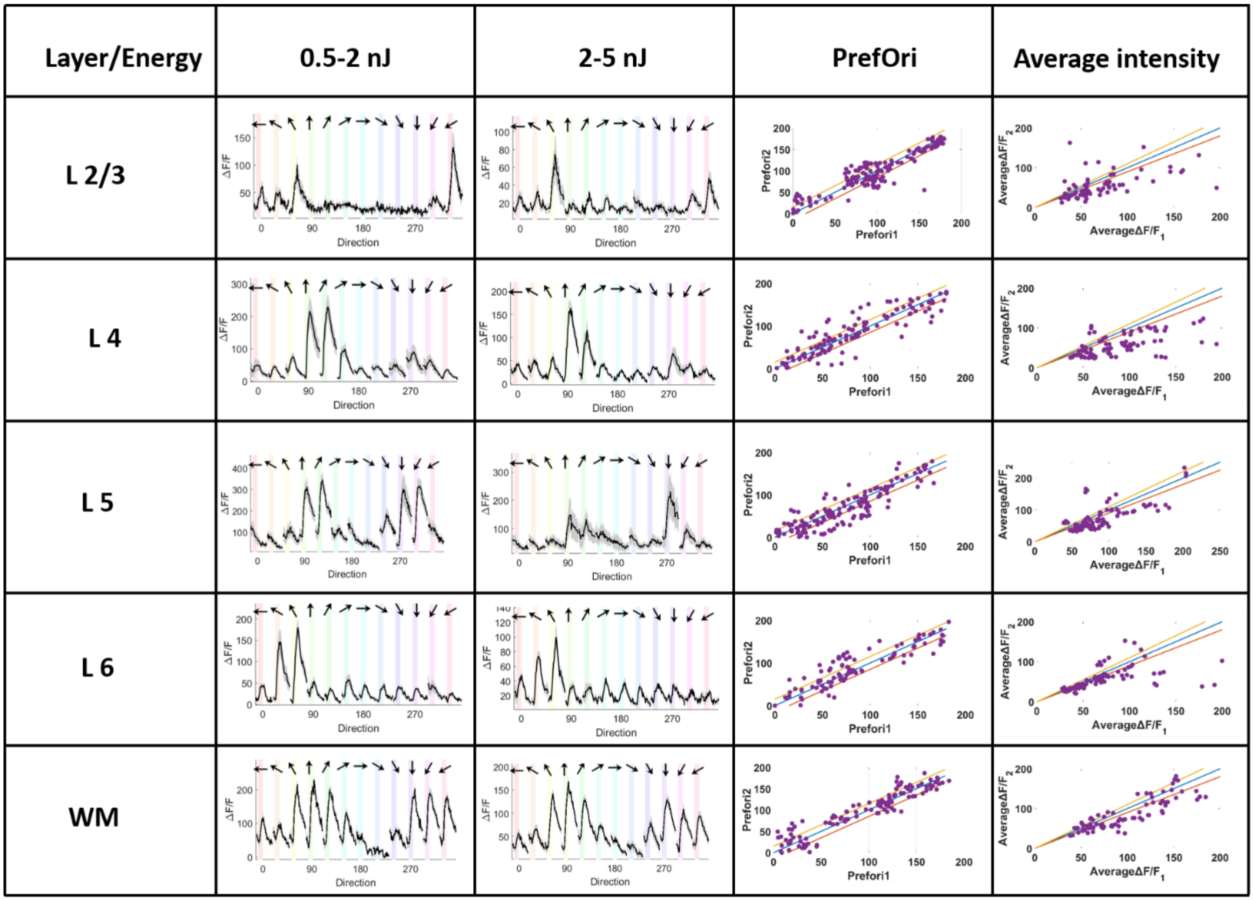


**Supplementary Figure 8. Comparison of evoked neuronal responses at each layer for two pulse energy categories.** Representative average evoked neuronal responses of the same cell in each layer for pulse energy range between 0.5-2 nJ (first column) and 2-5 nJ (second column). Preferred orientation of each cell at each layer for these two pulse energy conditions (third column). Blue color represents the condition where both preferred orientations are the same, yellow and orange colors represent conditions where the preferred orientation differs by 15 degrees. Average response at the preferred orientation for each cell at these two pulse energies (fourth column). Blue line represents the condition where responses are the same, yellow and orange lines represent conditions where responses differ by 10%.

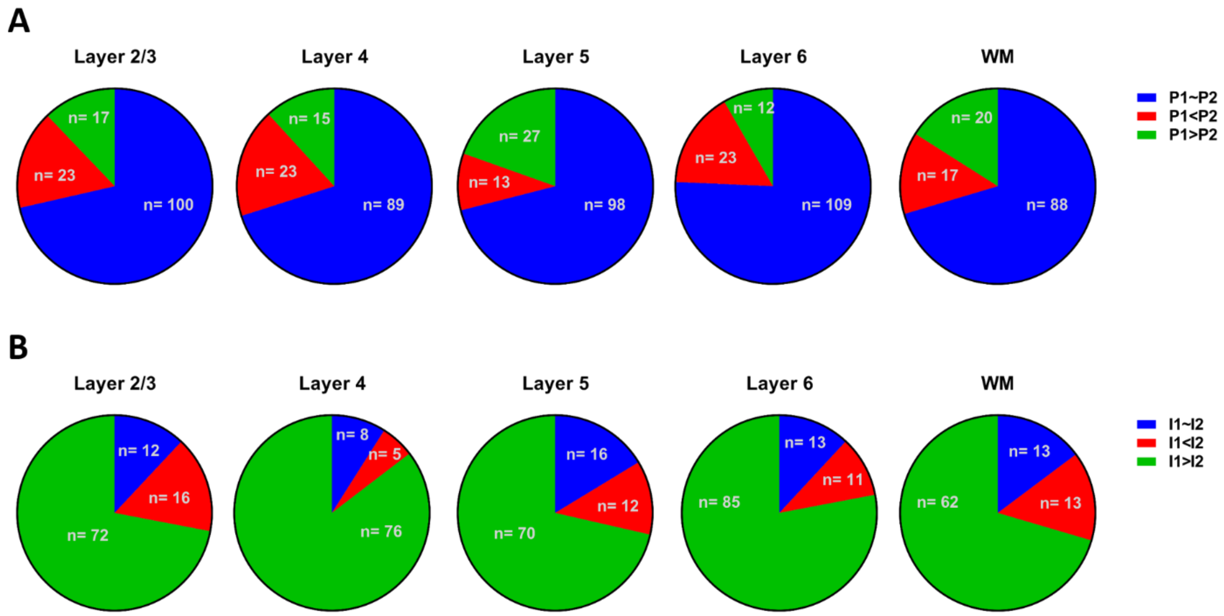


**Supplementary Figure 9. Comparison of preferred orientation and response at preferred orientation of neuronal populations in each layer at two different pulse energy groups**. A. Comparison of preferred orientation of neuronal populations across layers at energy level 1 (P1, first column in Supp.Fig. 8) and energy level 2 (P2, second column in Supp. Fig. 8). B. Comparison of average response at preferred orientation across each layer at energy level 1 (I1) and energy level 2 (I2). Three animals were used for this comparison, n represents overall number of cells.

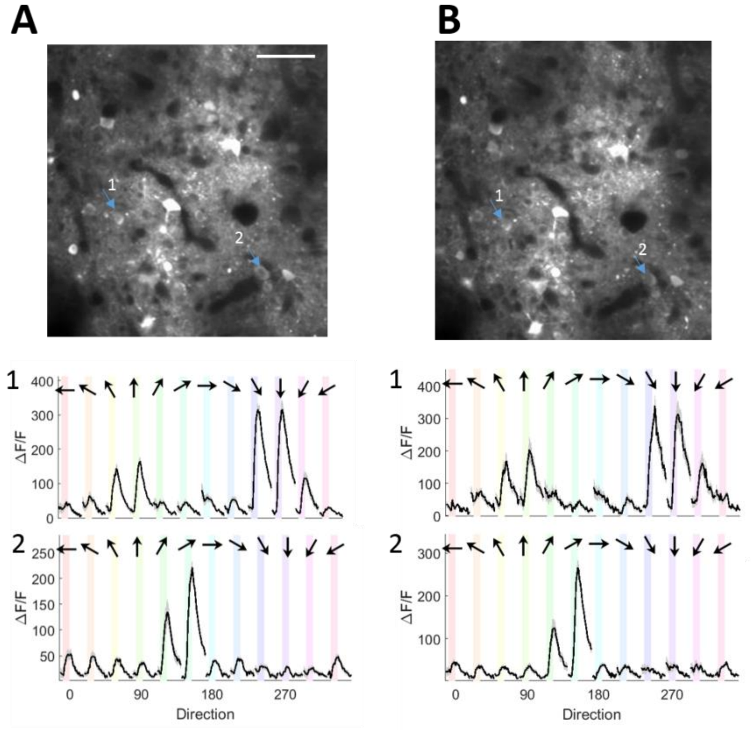


**Supplementary Figure 10. Comparison of evoked neuronal responses of L2/3 cells with 3p microscopy before and after imaging deep layers**. (A) Representative image of L2/3 cells and their responses to sinusoidal gratings while imaging them for the first time. (B) Reimaging of the same cells after imaging deep layers, showing responses and tuning to sinusoidal gratings.

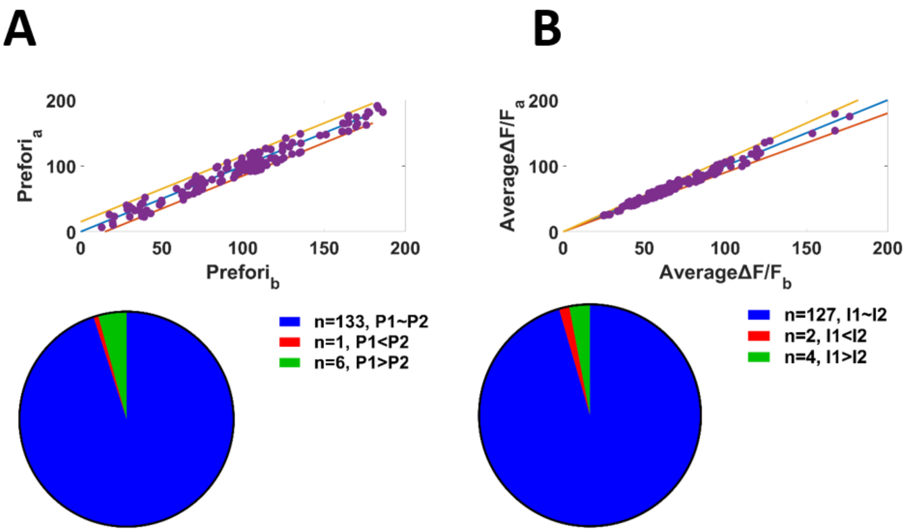


**Supplementary Figure 11. Population analysis of L2/3 cells, for their preferred orientation and peak fluorescence intensity before and after imaging deep layers**. (A) Comparison of preferred orientation of L2/3 cells before (subscript b) and after (subscript a) imaging deep layers (top; n=3 animals, 140 cells). Blue line represents the condition where both preferred orientations are the same, yellow and orange lines represent conditions where preferred orientation differs by 15 degrees; 95% of cells share similar preferred orientations (bottom). (B) Comparison of responses at the preferred orientation of L2/3 cells which share their preferred orientation (n=133 cells, from A). Blue line represents the condition where responses are the same, yellow and orange lines represent conditions where responses differ by 10%; 95% of cells share similar responses (bottom).

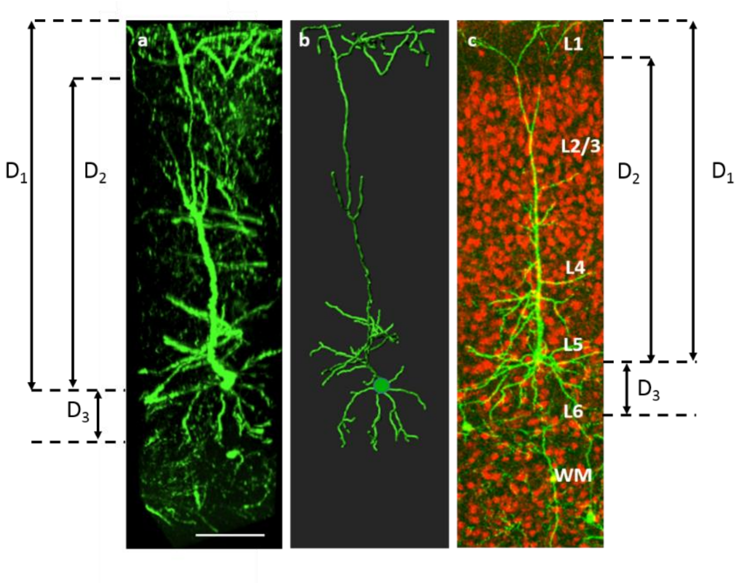


**Supplementary Figure 12. Three-photon microscope imaging of layer 5 pyramidal neuron in awake GFP-M mice.** (a) Three-photon image of a layer 5 neuron (550 µm depth) showing its apical and basal dendrites. (b) Model of the neuron in (a), including the somata and apical and basal dendrites, rendered with Imaris software. (c) Histological section of V1 in a GFP-M mouse highlighting a similar neuron. NeuN staining (red) is used to identify each layer in the cortex. Scale bar in (a) represents 100 µm.

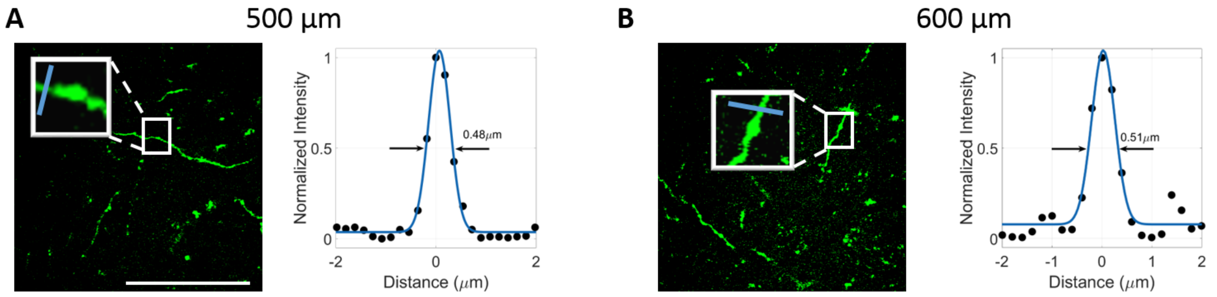


**Supplementary Figure 13. Resolution characterization of three-photon imaging of layer 5 pyramidal neuron in awake GFP-M mice.** (A) Three-photon image of a layer 5 neuron 50 µm above the somata of the neuron (left); linear profile of the intensity in the dendrite (right). (B) Three-photon image of the same neuron 50 µm below the somata of the neuron (left); linear profile of the intensity in the dendrite (right). Scale bar is 100 µm.

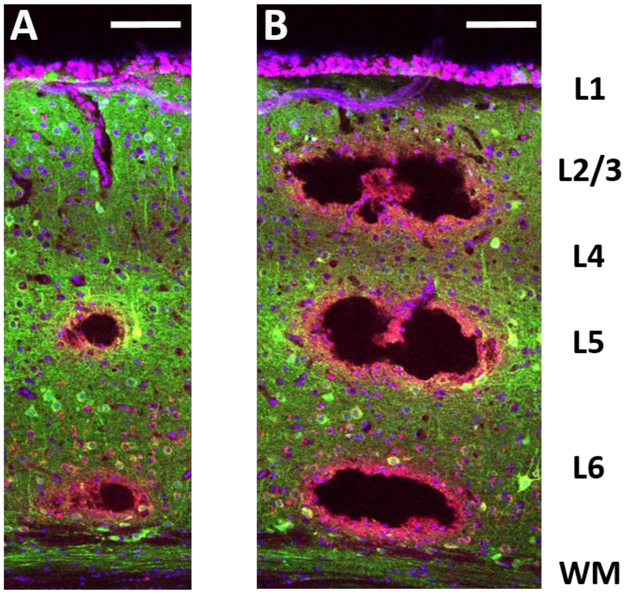


**Supplementary Figure 14. Creating laser ablation markers at multiple depths in the visual cortex.** Laser ablation markers were created with the pump laser (1045 nm) at 250, 500, and 750 µm depths with (A) pulse energies of 250 nJ, 500 nJ, and 1 µJ, and (B) 1 µJ, 2 µJ, and 4 µJ (B), respectively. Lower pulse energies resulted in smaller ablations (A) compared to (B). [In A, due to the large blood vessel in layer 2/3, no ablation marker was observed at 250 µm depth]. DAPI (blue), GCaMP 6s (green), and retrobeads (red) labeling was overlaid. Retrobeads labeling of cortical neurons projecting to the visual thalamus helped to determine layer 6. Low and high level of GCaMP6s activity revealed layer 4 and 5, respectively. Small size and high number of cells with DAPI staining helped to determine layer 2/3. White matter (WM) was evident as transverse fibers. Scale bar represents 100 µm.

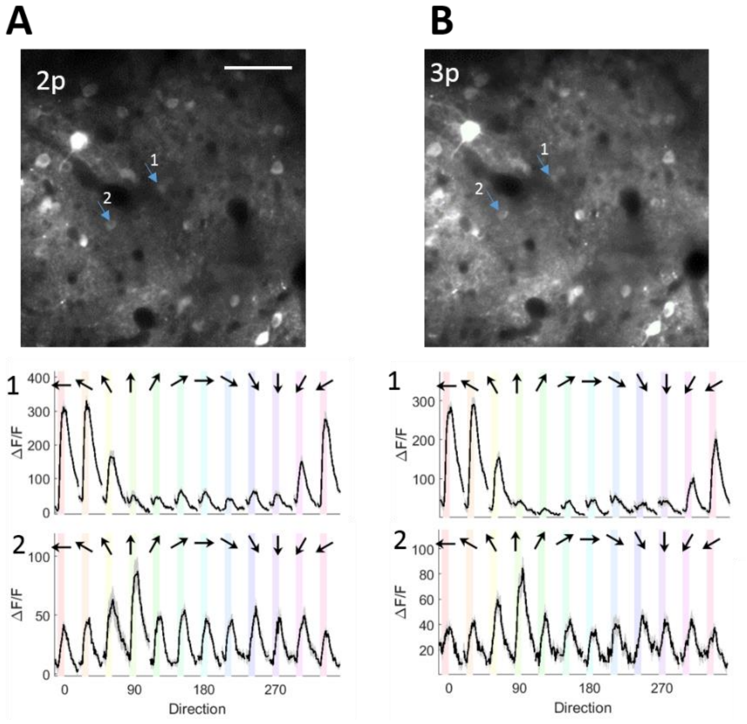


**Supplementary Figure 15. Comparison of evoked neuronal responses of L2/3 cells with 2p and 3p microscopy**. (A) Representative image of L2/3 cells and their tuning properties to sinusoidal gratings with 2p microscopy. (B) Reimaging the same cells with 3p microscopy. Neurons 1 and 2 are marked by arrows in the 2p and 3p images.

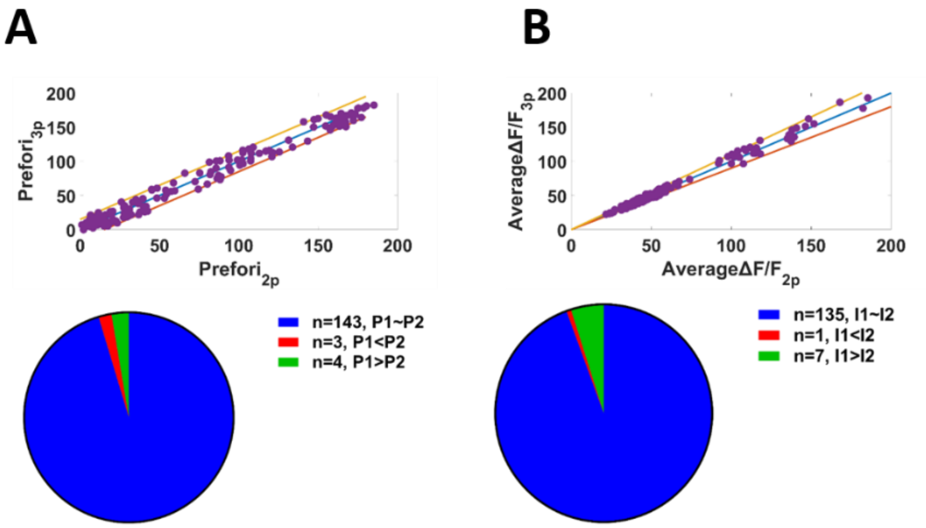


**Supplementary Figure 16. Population analysis of the same L2/3 cells for their preferred orientation and peak fluorescence intensity with 2p and 3p microscopy**. (A) Comparison of preferred orientation of L2/3 cells with 2p and 3p microscopy (top; n=3 animals, 150 cells). Blue line represents the condition where both preferred orientations are the same, yellow and orange lines represent the condition where preferred orientation differs by 15 degrees; 95% of cells share similar preferred orientations (bottom). (B) Comparison of responses at the preferred orientation of L2/3 cells which share their preferred orientation (n=143 cells, from A). Blue line represents the condition where responses are the same, yellow and orange lines represent the conditions where responses differ by 10%; 94% of cells share similar responses (bottom).

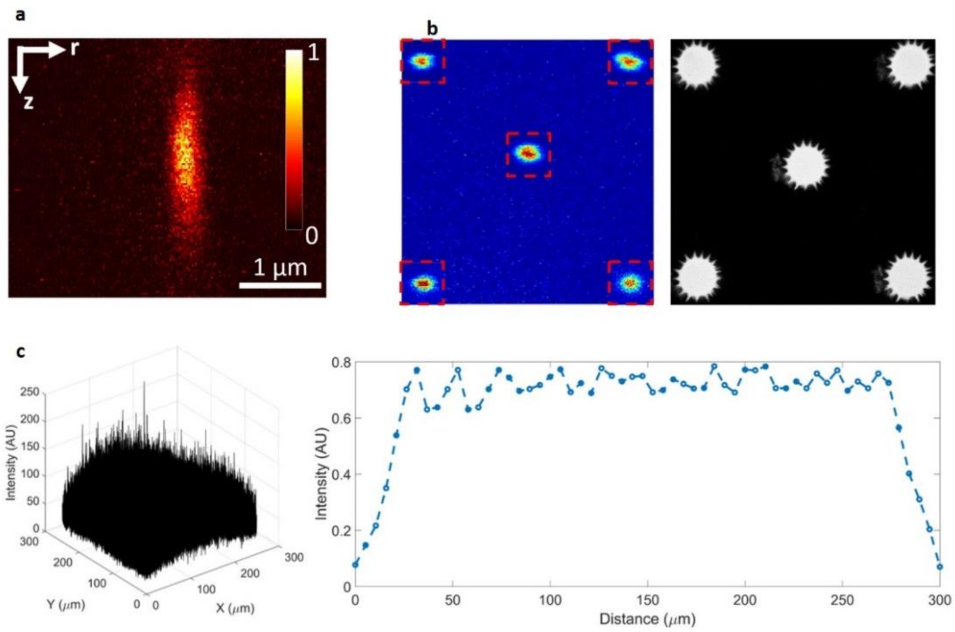


**Supplementary Figure 17. Characterization of microscope performance**. (a) Two-dimensional (2-D) point spread function (PSF) of a representative fluorescent bead. The lateral and axial PSF is measured as 0.45 ± 0.05 µm and 1.9 ± 0.2 µm, respectively. Errors represent the standard error of the mean. The corresponding effective numerical aperture is 0.9. (b) Excitation uniformity test with fluorescent beads (left) and pollen grains (right). For fluorescent bead imaging, the fluorescent beads on the corners and in the middle were chosen and images were collected at 10 different locations. To make them more visible, fluorescent beads were zoomed. For pollen grain imaging, a spiky pollen grain was chosen and translated on the 4 corners and in the middle of the field of view and imaged with the sample laser power. (c) Rhodamine 6G dye was deposited on a glass slide and imaged on the GCaMP PMT. 2-D intensity profile of this image is shown on the left. 1-D intensity profile was plotted to determine the field of view with uniform intensity profile on the right.

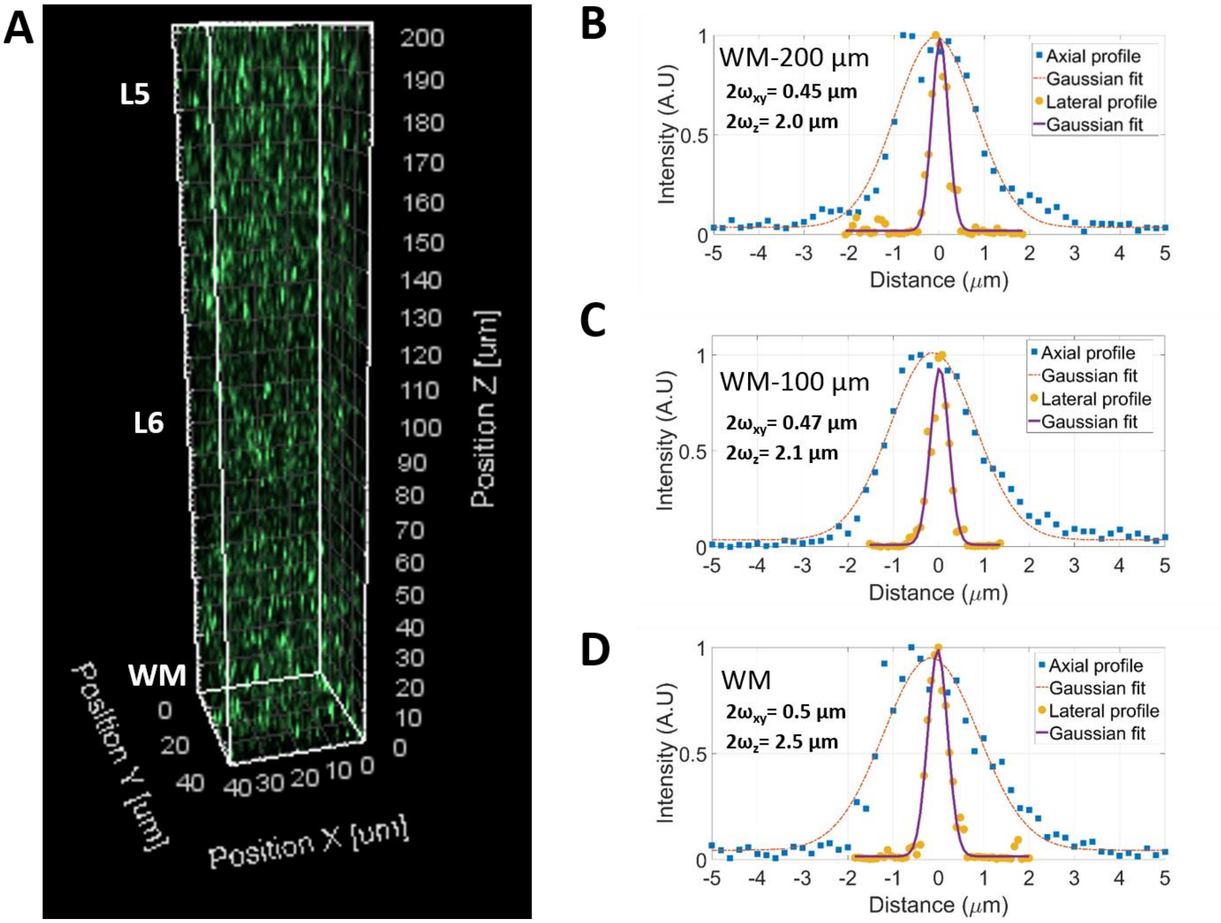


**Supplementary Figure 18. In vivo point spread function (PSF) utilizing green retrobeads in anesthetized mice.** (A) 3D rendering of 3p imaging of retrobeads in a 200 µm column within deep cortex, spanning L6 and extending into L5 and the top of WM. Lateral and axial PSF profile for 200 um above WM (B), 100 um above WM (C) and at the boundary of WM and L6 (D). Lateral and axial point spread function values are given in the inset of panels B, C, and D.

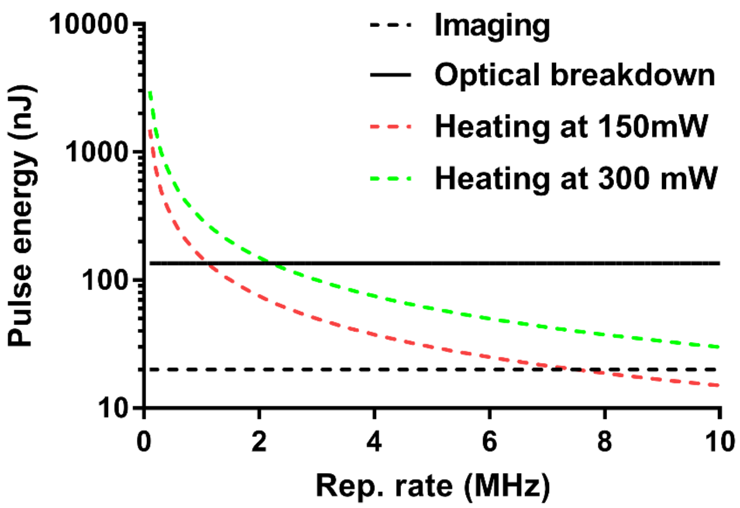


**Supplementary Figure 19. Comparing pulse energy for optical breakdown, bulk heating, and three-photon imaging at 600 μm (layer 6) in mouse visual cortex at 40 fs pulse width.** Three-photon imaging requires 10-20 nJ pulse energy to image the entire depth of cortex and this pulse energy requirement is independent of the repetition rate. Similarly, threshold energy for optical breakdown is also independent of the repetition rate, and has a value between 100-150 nJ. However, bulk heating depends on average laser power so that required pulse energy is dependent on the repetition rate. We show curves for two different threshold power values (150 and 300 mW) for bulk heating (see text). For moderate repetition rates (<1 MHz, 150 mW curve), optical breakdown initiates laser damage, whereas bulk heating dominates for higher repetition rates (>2 MHz, 300 mW curve). For moderate repetition rates (<1 MHz), threshold energy for 3-photon imaging is at least 5-10 fold smaller than required energy for optical breakdown and bulk heating. However, for higher repetition rates (>2 MHz), threshold energies for 3-photon imaging and bulk heating get closer to each other and increase the risk of laser damage during imaging.

**Supplementary Table 1. Parameters for the designed objective**

| | |
|---|---|
| Working distance (mm) | 2.05 |
| Overall length (mm) | 80.5 |
| Overall transmission (%) | 70 |
| Effective focal length (mm) | 7.2 |
| Clear aperture (mm) | 15 |
| 1-photon FWHM lateral resolution (µm) | 0.8 |
| Numerical aperture | 1.02 |

**Supplementary Table 2. Quantification of three-photon and histology images from GFP-M animal**

| | $D_1$ (µm) | $D_2$ (µm) | $D_3$ (µm) |
|---|---|---|---|
| 3p | 544 | 455 | 72 |
| Histology | 500 | 449 | 75 |

**Supplementary Table 3. Number of Orientation Selective (OS) neurons across all layers of the visual cortex and white matter**

| **Layer/Animal** | **Animal 1** | **Animal 2** | **Animal 3** | **Animal 4** | **Animal 5** | **Total** |
|---|---|---|---|---|---|---|
| **L2/3** | 54 (68%) | 52 (72%) | 45 (73%) | 50 (75%) | 40 (68%) | **241** |
| **L4** | 44 (81%) | 46 (82%) | 38 (79%) | 42 (79%) | 40 (85%) | **210** |
| **L5** | 46 (87%) | 39 (95%) | 54 (92%) | 37 (90%) | 38 (88%) | **214** |
| **L6** | 47 (85%) | 44 (85%) | 39 (87%) | 42 (89%) | 37 (86%) | **209** |
| **WM** | 43 (45%) | 48 (48%) | 38 (44%) | 35 (42%) | 41(48%) | **205** |
| **Total** | **234** | **229** | **214** | **206** | **196** | **1079** |

**Supplementary Table 4. Mean values and standard error of the mean (SEM) of visual parameters across all layers of the visual cortex and white matter**

| | gOSI | OSI | DSI |
|---|---|---|---|
| **L2/3** | 0.45±0.01 | 0.72±0.02 | 0.38±0.02 |
| **L4** | 0.44±0.02 | 0.76±0.02 | 0.38±0.02 |
| **L5** | 0.34±0.01 | 0.65±0.02 | 0.34±0.02 |
| **L6** | 0.49± 0.02 | 0.71±0.02 | 0.38±0.02 |
| **WM** | 0.25 ± 0.01 | 0.44 ± 0.02 | 0.25±0.01 |

**Supplementary Table 5. P-values of one-way ANOVA test with multiple comparisons for gOSI values for each layer**

| | L2/3 | L4 | L5 | L6 | WM |
|---|---|---|---|---|---|
| **L2/3** | NA | >0.9999 | <0.0001 | 0.0387 | <0.0001 |
| **L4** | >0.9999 | NA | <0.0001 | 0.0467 | <0.0001 |
| **L5** | <0.0001 | <0.0001 | NA | <0.0001 | <0.0001 |
| **L6** | 0.0387 | 0.0467 | <0.0001 | NA | <0.0001 |
| **WM** | <0.0001 | <0.0001 | <0.0001 | <0.0001 | NA |

**Supplementary Table 6. P-values of one-way ANOVA test with multiple comparisons for OSI values for each layer**

| | L2/3 | L4 | L5 | L6 | WM |
|---|---|---|---|---|---|
| **L2/3** | NA | 0.0833 | 0.0121 | 0.9974 | <0.0001 |
| **L4** | 0.0833 | NA | <0.0001 | 0.0685 | <0.0001 |
| **L5** | 0.0121 | <0.0001 | NA | 0.0280 | <0.0001 |
| **L6** | 0.9974 | 0.0685 | 0.0280 | NA | <0.0001 |
| **WM** | <0.0001 | <0.0001 | <0.0001 | <0.0001 | NA |

**Supplementary Table 7. P-values of one-way ANOVA test with multiple comparisons for DSI values for each layer**

| | L2/3 | L4 | L5 | L6 | WM |
|---|---|---|---|---|---|
| **L2/3** | NA | 0.8225 | 0.0148 | 0.9228 | <0.0001 |
| **L4** | 0.8225 | NA | 0.0153 | >0.9999 | <0.0001 |
| **L5** | 0.0148 | 0.0153 | NA | 0.0284 | <0.0001 |
| **L6** | 0.9228 | >0.9999 | 0.0284 | NA | <0.0001 |
| **WM** | <0.0001 | <0.0001 | <0.0001 | <0.0001 | NA |

**Supplementary Table 8. Point Spread Function measurement with fluorescent beads at 5 different locations (errors represent standard error of the mean)**

| Location of the bead | Lateral resolution (µm) | Axial resolution (µm) |
|---|---|---|
| Top left corner | 0.43 ± 0.07 | 1.82 ± 0.12 |
| Top right corner | 0.48 ±0.05 | 1.92 ±0.11 |
| Bottom left corner | 0.46 ±0.07 | 1.86 ±0.11 |
| Bottom right corner | 0.46 ±0.06 | 1.92 ±0.10 |
| Center | 0.44 ±0.05 | 1.89 ±0.15 |

**Supplementary Table 9. Three-photon signal intensity distribution across pollen grains at 5 different locations (errors represent standard error of the mean)**

| Location of the pollen grain | Mean Signal intensity (A.U.) |
|---|---|
| Top left corner | 55933 ± 1876 |
| Top right corner | 56001 ± 1448 |
| Bottom left corner | 56127 ± 2160 |
| Bottom right corner | 56174 ± 2188 |
| Center | 55260 ± 1500 |
